# Whole blood gene expression in infants with respiratory syncytial virus bronchiolitis

**DOI:** 10.1186/1471-2334-6-175

**Published:** 2006-12-13

**Authors:** Hans-Olav Fjaerli, Geir Bukholm, Anne Krog, Camilla Skjaeret, Marit Holden, Britt Nakstad

**Affiliations:** 1 University of Oslo, Faculty Division Akershus University Hospital, Department of Paediatrics, Akershus University Hospital, Norway; 2 Institute of Clinical Epidemiology and Molecular Biology, Akershus University Hospital, Norway; 3 Norwegian Computing Center, Oslo, Norway

## Abstract

**Background:**

Respiratory syncytial virus (RSV) is a major cause of viral bronchiolitis in infants worldwide, and environmental, viral and host factors are all of importance for disease susceptibility and severity. To study the systemic host response to this disease we used the microarray technology to measure mRNA gene expression levels in whole blood of five male infants hospitalised with acute RSV, subtype B, bronchiolitis versus five one year old male controls exposed to RSV during infancy without bronchiolitis. The gene expression levels were further evaluated in a new experiment using quantitative real-time polymerase chain reaction (QRT-PCR) both in the five infants selected for microarray and in 13 other infants hospitalised with the same disease.

**Results:**

Among the 30 genes most differentially expressed by microarray nearly 50% were involved in immunological processes. We found the highly upregulated interferon, alpha-inducible protein 27 (IFI27) and the highly downregulated gene Charcot-Leyden crystal protein (CLC) to be the two most differentially expressed genes in the microarray study. When performing QRT-PCR on these genes IFI27 was upregulated in all but one infant, and CLC was downregulated in all 18 infants, and similar to that given by microarray.

**Conclusion:**

The gene IFI27 is upregulated and the gene CLC is downregulated in whole blood of infants hospitalised with RSV, subtype B, bronchiolitis and is not reported before. More studies are needed to elucidate the specificity of these gene expressions in association with host response to this virus in bronchiolitis of moderate severity.

## Text

Why some infants develop bronchiolitis when exposed to respiratory syncytial virus (RSV) is poorly understood, and several aspects of environmental and host immunity have been extensively studied [1,2]. The role of the virus itself in modulating host immune response has also been investigated. Recent studies have revealed an important role of certain RSV surface proteins to counteract the host interferon (IFN) response, a response known to be important for viral clearance [3,4].

Microarray can detect the simultaneous expressions and interactions of thousands of genes [5]. This technology was used as a hypothesis generating tool to identify the most differentially expressed genes in whole blood of five male infants hospitalised with RSV, subtype B, bronchiolitis versus five one year old male controls exposed to RSV during infancy without being hospitalized and/or treated for acute bronchiolitis. To further validate our results quantitative real-time polymerase chain reaction (QRT-PCR) was performed on the most differentially expressed genes given by the microarray experiment both in the infants selected for microarray and in a new population of 13 more infants admitted to our hospital with the same disease [6]. Four of the five one year old male controls were used as a pooled exogenous control in the QRT-PCR study.

The infants were diagnosed with bronchiolitis if having symptoms from the lower airways characterized by wheezing, dyspnea, respiratory distress, poor feeding, tachypnea and fine crepitations upon auscultation when examined by the doctor on call in the emergency room [7]. The doctor also conducted a structured clinical interview with the parents and NPA was taken for viral analysis with multiplex RT-PCR [8]. Median duration of symptoms prior to hospitalisation was 4 (range 2–7) days, median age at admission was 3 (range 1–8) months and median duration of hospitalisation was 3 (range 1–8) days. Severity of illness was comparable in all 18 infants, except for one infant who had a prolonged hospitalisation period of 8 days. However, all infants received the same routine treatment including inhalations with nebulised racemic epinephrine and none of the infants needed artificial ventilation. More information on the selection of cases and controls is described elsewhere [See Additional file 1].

### Microarray results

The Feature Extraction software identified 19663 unique gene sequences. After normalization and filtering of control spots and spots with signal intensities below 300 in either channel a total of 15761 genes were available for further evaluation. When performing statistical analysis of the data using the BAMarray software we found 439 genes to be significantly differentially expressed between cases and controls. Furthermore, by also analyzing the same data with the Rosetta Luminator software, a list of 1544 genes with P value ≤ 0.01 was identified. 54 of these genes had mean fold changes of at least ± 2.50. Finally, by comparing the list of 439 genes found by BAMarray with the list of 54 genes identified with the Rosetta Luminator software, a total of 30 genes occurred in both lists and were selected for final presentation (tab 1). More methodological details are described elsewhere [See Additional file 1].

**Table 1:** Significant genes with mean fold changes^1 ^of at least ± 2.50 as given by microarray^2^

GeneBank identifier	Gene symbol	Gene name	Biological process	Fold change
NM_005532	IFI27	Interferon, alpha-inducible protein 27	Immune response	+13.29
NM_005143	HP	Haptoglobin	Defence response	+5.71
NM_006417	IFI44	Interferon-induced protein 44	Immune response	+4.86
NM_024021	MS4A4A	Membrane-spanning 4-domains, subfamily A, member 4	Signal transduction	+4.42
NM_004994	MMP9	Matrix metalloproteinase 9	Collagen catabolism	+3.93
BC035682	HBZ	Hemoglobin, zeta	Oxygen transport	+3.78
NM_015535	DNAPTP6	DNA polymerase-transactivated protein 6		+3.57
NM_000519	HBD	Hemoglobin, delta	Oxygen transport	+3.40
NM_005330	HBE1	Hemoglobin, epsilon 1	Oxygen transport	+3.06
NM_006187	OAS3	2'-5'-oligoadenylate synthetase 3, 100kDa	Immune response	+2.99
NM_000566	FCGR1A	Fc fragment of IgG, high affinity Ia, receptor (CD64)	Immune response	+2.88
NM_006949	STXBP2	Syntaxin binding protein 2	Intracellular protein transport	+2.86
NM_033255	EPSTI1	Epithelial stromal interaction 1 (breast)		+2.83
NM_002759	EIF2AK2	Eukaryotic translation initiation factor 2-alpha kinase 2	Immune response	+2.78
NM_016633	ERAF	Erythroid associated factor	Hemoglobin metabolism	+2.73
NM_006770	MARCO	Macrophage receptor with collagenous structure	Response to pathogenic bacteria	+2.72
NM_001724	BPGM	2,3-bisphosphoglycerate mutase	Carbohydrate metabolism	+2.60
NM_000904	NQO2	NAD(P)H dehydrogenase, quinone 2	Electron transport	+2.56
NM_006820	IFI44L	Interferon-induced protein 44-like	Immune response	+2.55
NM_005101	G1P2	Interferon, alpha-inducible protein (clone IFI-15K)	Immune response	+2.54
NM_024572	GALNT14	UDP-N-acetyl-alpha-D-galactosamine:polypeptide N-acetylgalactosaminyltransferase 14		+2.53
NM_203339	CLU	Clusterin	Immune response	+2.50
NM_005581	LU	Lutheran blood group (Auberger b antigen included)	Cell adhesion	-2.55
NM_080819	GPR78	G protein-coupled receptor 78	G-protein coupled receptor protein signaling pathway	-2.70
NM_000797	DRD4	Dopamine receptor D4	G-protein coupled receptor protein signaling pathway	-2.89
NM_006249	PRB3	Proline-rich protein BstNI subfamily 3	G-protein coupled receptor protein signaling pathway	-2.91
NM_021983	HLA-DRB4	Homo sapiens major histocompatibility complex, class II, DR beta 4	Immune response	-3.31
NM_000558	HBA1	Hemoglobin alpha 1	Oxygen transport	-4.61
L34088	HLA-DQA1	Major histocompatibility complex, class II, DQ alpha 1	Immune response	-4.70
NM_001828	CLC	Charcot-Leyden crystal protein	Antimicrobial humoral response	-6.08

^1^Gene expressions given as mean of the individual fold changes (signal intensity in a case minus signal intensity in the corresponding control). ^2^Gene expressions as given by microarray in whole blood of five male infants hospitalised with respiratory syncytial virus, subtype B, bronchiolitis versus five one year old male controls exposed to RSV during infancy but not hospitalised and/or treated for bronchiolitis

22 of these 30 genes were upregulated while 8 genes were downregulated, and 13 of the 30 genes were involved in several aspects of immune response. Among these were the upregulated interferon inducible genes IFI27, IFI44, EIF2AK2, IFI44L, OAS3 and G1P2 and the downregulated genes HLA-DRB4, HLA-DQA1 and CLC (tab 1).

### QRT-PCR results

A QRT-PCR study with TaqMan Low Density Array (TLDA) cards was performed on 23 of the most differentially expressed genes given by microarray to compare the two methods before the study was applied on a new patient population. Six genes failed amplification and were excluded from further analyses. More methodological details are described elsewhere [See Additional file 1].

**Figure 1:**
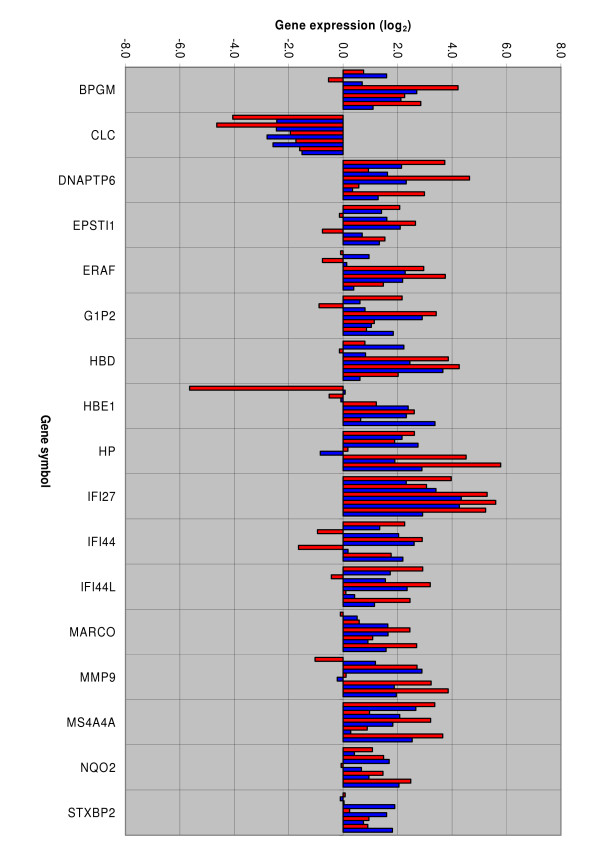
**Evaluation of genes differentially expressed by microarray (blue bars) with QRT-PCR (red bars)**. The microarray experiment was conducted as a case-control study with whole blood gene expressions given as ratio of signal intensity in each of five male infants hospitalised with respiratory syncytial virus, subtype B, bronchiolitis versus signal intensity in a corresponding one year old male control exposed to the virus during infancy but not hospitalised and/or treated for bronchiolitis. The quantitative real-time polymerase chain reaction (QRT-PCR) study used TaqMan Low Density Array (TLDA) cards with gene expression given as mean relative quantification (RQ) from triplets of each gene and analyzed in the same five male infants as selected for microarray. A pooled sample from four of the five one year old male controls was used as exogenous control and beta-glucuronidase (GUSB) was used as endogenous control in the QRT-PCR study. The following genes (gene symbol and name) were evaluated; BPGM: 2,3-bisphosphoglycerate mutase, CLC: Charcot-Leyden crystal protein, DNAPTP6: DNA polymerase-transactivated protein 6, EPSTI1: Epithelial stromal interaction 1 (breast), ERAF: Erythroid associated factor, G1P2: Interferon, alpha-inducible protein (clone IFI-15K), HBD: Hemoglobin, delta, HBE1: Hemoglobin, epsilon 1, HP: Haptoglobin, IFI27: Interferon, alpha-inducible protein 27, IFI44: Interferon-induced protein 44, IFI44L: Interferon-induced protein 44-like, MARCO: Macrophage receptor with collagenous structure, MMP9: Matrix metalloproteinase 9, MS4A4A: Membrane-spanning 4-domains, subfamily A, member 4, NQO2: NAD(P)H dehydrogenase, quinone 2, STXBP2: Syntaxin binding protein 2

Five of the 17 genes available for further evaluation (DNAPTP6, HP, IFI27, MS4A4A and STXBP2) were upregulated and one gene (CLC) was downregulated in all five male infants when compared to the pooled exogenous control. Seven genes (BPGM, G1P2, HBD, IFI44L, MARCO, MMP9 and NQO2) were upregulated in all but one infant while four genes (EPSTI1, ERAF, HBE1 and IFI44) were upregulated in all but two infants (fig 1). When comparing the gene expression results of the TLDA study and the microarray experiment for each of the five infants the expression profiles were quite similar for the genes CLC, DNAPTP6, IFI27 and MS4A4A. The remaining genes showed more variations (fig 1).

**Figure 2:**
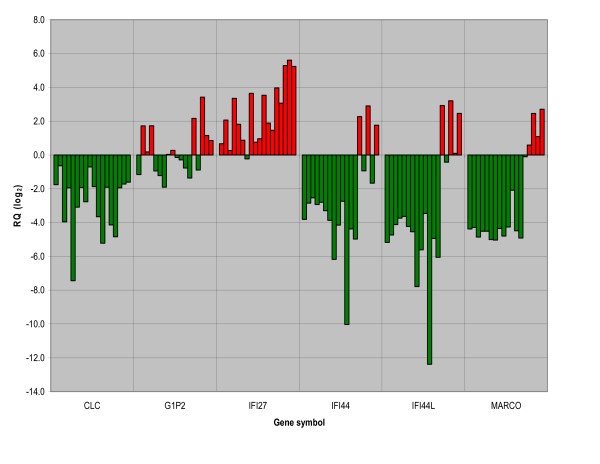
**QRT-PCR study of six whole blood immune response genes as given by microarray**. A quantitative real-time polymerase chain reaction (QRT-PCR) study using TaqMan Low Density Array (TLDA) cards was performed with gene expressions given as mean relative quantification (RQ) from triplets of each gene. Analyzed in 18 infants hospitalised with respiratory syncytial virus, subtype B, bronchiolitis versus a pooled sample from four one year old male children exposed to RSV during infancy but not treated and/or hospitalised for bronchiolitis during infancy as exogenous control and with beta-glucuronidase (GUSB) as endogenous control. The following genes (gene symbol and name) were studied; CLC: Charcot-Leyden crystal protein, G1P2: Interferon, alpha-inducible protein (clone IFI-15K), IFI27: Interferon, alpha-inducible protein 27, IFI44: Interferon-induced protein 44, IFI44L: Interferon-induced protein 44-like, MARCO:Macrophage receptor with collagenous structure

To evaluate the impact of the 17 genes expressed by microarray a new cohort was tested using QRT-PCR. In the new cohort of 13 infants hospitalised with the same disease 11 of the 17 genes were significantly differentially expressed in accordance with the results from the microarray study (p < 0.01; independent samples t-test). In order to see if some of the genes associated with immunological processes could also be differentially expressed in the new cohort, as well as in the five infants selected for microarray, we evaluated the expressions of the six immune response genes available for studies with TLDA cards. The gene IFI27 was upregulated in 17 infants and slightly downregulated in one infant while the gene IFI44 was downregulated in 15 infants and upregulated in three infants. The genes IFI44L and MARCO were downregulated in 14 infants and upregulated in four infants, and the gene G1P2 was downregulated and upregulated in nine infants, respectively. Finally, the gene CLC was downregulated in all 18 infants (fig 2).

## Discussion

The aim of the present study was to use microarray as a hypothesis generating tool to identify the most differentially expressed genes in whole blood of infants hospitalised with RSV, subtype B, bronchiolitis, and to further evaluate some of these genes with QRT-PCR. We found the interferon, alpha-inducible protein 27 (IFI27) and Charcot-Leyden crystal protein (CLC) to be the most differentially expressed genes in this study and not to be reported before in conjunction with acute viral bronchiolitis.

All 18 hospitalised infants in our study suffered from acute bronchiolitis of moderate severity. They were hospitalised because of severe respiratory difficulties, but were not in need of assisted ventilation including treatment with continuous positive airway pressure (CPAP) or mechanical ventilation. Our population is therefore representative for the majority of cases hospitalised during infancy worldwide [9]. The children in the control group were all healthy one year old children at the time of clinical examination and blood collection. They were representing the majority of infants undergoing RSV infection with only mild symptoms. This group was chosen to exclude children from the control group that could have a possible genomic predisposition for a more abnormal immune response, characterizing the infants with overt bronchiolitis. We would also be interested in differentiating between the gene expression profile of mild cases and severe cases of RSV infection, but this will require a new study and a design specific to support this question. The gene expression profile found in the present study is the result of RSV infection. It would in further studies be interesting to examine if other respiratory viral infections causing bronchiolitis give a similar or different expression pattern.

One limitation of the present study is the discrepancy in age between the cases and controls. It is well known that the immunological responses mature very rapidly from birth to age one year and the differences observed may therefore simply be due to age rather than to any infection related host response [10]. In order to confirm our gene expression results new studies, which also include a control group being more closely age matched, are very much needed. This study is also rather small, consisting of only 18 infants, and warrants us to be cautious to the interpretations of the results given.

The immune response to RSV infection, as with any other infection is comprised of an innate response, and subsequently by activation of humoral and cellular specific immunity. The cellular immune response seems to be important in controlling the infection once initiated and for clearance of the virus, while the humoral immune response is important in providing protection against subsequent infection [11]. The interferons (IFNs) are a diverse family of cytokines consisting in humans mainly of IFN-alpha and IFN-beta (type I) and IFN-gamma (type II). Both types play an essential role in host immunity by inhibiting the replication and spread of viral, bacterial, and parasitic pathogens. Several studies have shown the importance of IFN-gamma as regulators of host response to RSV infection [12,13]. However, the sensitivity of RSV to the antiviral activity of INF-alpha is known to be low, at least when compared with that of other viruses [14]. IFNs mediate their effects via transcription of interferon stimulated genes (ISGs) by binding to cell surface receptors activating members of the JAK-STAT pathway [15]. Studies have shown an important role of the RSV surface protein NS1 to antagonize the type I IFN-mediated antiviral response [16]. Also, recent in vitro studies have shown that knocking out the NS1 gene results in upregulation of several ISGs, increased viral clearance and modulation of the host response towards a Th1 phenotype [3,4,17]. The gene IFI27 belong to a group of small ISGs [18,19]. Its biological functions have yet to be revealed, but studies have found this gene to be highly upregulated during cancer development in several tissues and in children with untreated juvenile dermatomyositis [20]. It has also been shown to have a direct antiviral effect against certain viruses [21]. In our study IFI27 was highly upregulated in all but one infant even after evaluation with QRT-PCR. However, the specific biological role(s) of this gene, if any, in early-life RSV bronchiolitis need more studies.

Major basic protein (MBP), eosinophil peroxidase (EPO), eosinophil cationic protein (ECP), eosinophil-derived neurotoxin (EDN) and Charcot-Leyden crystal (CLC) protein are major secretory effector proteins of eosinophils [22]. Eosinophilia of blood and tissue is classically associated with parasitic and especially invasive helminthic infections and is not typical for either bacterial or viral infections. However, several groups have shown evidence of eosinophil degranulation in the lung parenchyma during RSV infection, and both ECP and EDN are shown to have antiviral effects against RSV in vitro [23]. Furthermore, in vivo studies of infants with RSV bronchiolitis have found blood levels of ECP to be higher during convalescence than during acute disease and therefore possibly play a role in the development of the long-standing inflammatory reaction seen in the airways after RSV bronchiolitis, and in which eosinophils play an important role [24,25]. In our study we found the gene coding for the eosinophil-derived phospholipase CLC to be downregulated in peripheral blood of all infants even after evaluation with QRT-PCR. Studies have shown that eosinophil-derived phospholipases may contribute to surfactant dysfunction in the asthmatic lung [26]. However, the possible importance of CLC downregulation in peripheral blood of infants with RSV bronchiolitis need more studies.

The present study shows the importance of further evaluation of microarray data with other methods such as QRT-PCR and protein expression analyses. Microarray and QRT-PCR are suitable tools for gene expression studies related to acute RSV bronchiolitis in infants and can increase our knowledge of the host response to this disease. The present study identified the genes IFI27 and CLC to be highly differentially expressed in whole blood of infants hospitalised with RSV, subtype B, bronchiolitis of moderate severity. However, more studies are needed to identify the specificity of these two genes in relation to this disease.

## Competing interests

The author(s) declare that they have no competing interests.

## Authors' contributions

HOF had primary responsibility for protocol development, outcome assessment, data acquisition and analyses and writing of the manuscript. BN and GB participated in the development of the protocol and analytic framework of the study, and contributed to the writing of the manuscript. AK and CS had primary responsibility for the laboratory work but participated also in the data analyses and the writing of the manuscript. MH took part in the statistical analysis of the microarray data and writing of the manuscript. All authors read and approved the final manuscript.

## Pre-publication history

The pre-publication history for this paper can be accessed here:



## Supplementary Material

Additional File 1 Materials and methods. The file contains a description of the selection of cases and controls, ethics, how we designed and performed the microarray experiment and the QRT-PCR study and some additional references. Click here for file
