# Supplementary material for: Whole blood gene expression in infants with respiratory syncytial virus bronchiolitis
Source: BMC Infect Dis. 2006 Dec 13;6:175. doi: 10.1186/1471-2334-6-175 (PMC1713240; doi:10.1186/1471-2334-6-175)
Supplement: Additional File 1 — Materials and methods. The file contains a description of the selection of cases and controls, ethics, how we designed and performed the microarray experiment and the QRT-PCR study and some additional references. [file 1471-2334-6-175-S1.doc]

# Materials and methods

**Selection of cases and controls**

From a total of 3500 deliveries in Akershus University Hospital during the period January 20^th^ 2002 to February 15^th^ 2003 a basic birth cohort of 2108 healthy term infants was established. If hospitalised with acute bronchiolitis during infancy a venous blood sample for isolation of total RNA and a sample of mucous from nasopharyngeal aspirate (NPA) for microbiological diagnosis were collected as part of the admission routine. 57 patients from the basic birth cohort were hospitalised with acute viral bronchiolitis during infancy. 27 of these infants suffered from bronchiolitis due to RSV, subtype B, and were selected for further evaluation. After performing laboratory tests on RNA quality, the results were acceptable for 18 of these 27 infants [1]. Blood samples from five randomly selected boys were then prepared for microarray analysis while the remaining 13 infants took part in the QRT-PCR study.

Based on a random selection from the basic birth cohort and preliminary telephone interview with the parents we also invited 10 boys who had not been hospitalised and/or treated for acute bronchiolitis during infancy to a follow-up visit at age one year. A blood sample was taken for isolation of total RNA and the parents were once again asked if the child had been hospitalised and/or treated for acute bronchiolitis during infancy. The answers were controlled by hospital discharge records. The interviews with the parents and clinical examinations of the children were all performed by the principal investigator (HOF). Eight boys tested positive to previous RSV exposure when analyzed for RSV antibodies with a standard complement fixation test [2]. Based on the laboratory tests on RNA quality, five boys were then selected as controls in the microarray experiment and a pooled RNA sample from four of these five boys was used as exogenous control in the QRT-PCR study.

**Ethics**

The study was approved by the Regional Research Ethics Committee (RREC), region East. Oral and written informed consent for further studies were given by the parents both at birth and when the child was hospitalised with acute bronchiolitis during infancy or when attending the follow-up visit at age one year for the controls.

**Microarray experiment**

Blood samples were collected in PaxGene RNA collection tubes (PreAnalytiX) according to the manufacturers recommendations and stored at -80° Celsius until RNA extraction [3]. Total RNA was extracted from the PaxGene RNA collection tubes using PaxGene blood RNA isolation kit (PreAnalytiX). RNA quality was determined with the Agilent 2100 Bioanalyzer (Agilent Technologies). Fluorescent complimentary RNA (cRNA) was generated by RNA amplification using the Agilent Low Input Linear Amplification kit (Agilent Technologies) following the manufactures descriptions, and each sample was labeled with either Cyanine (Cy) 3 or Cy5 (Perkin-Elmer). The final cRNA concentration was determined with the ND-1000 spectrophotometer (NanoDrop Technologies).

A dye-swapped case-control experimental design was chosen where each of the five dye-swaps consisted of samples from one patient and one control. 1 μg of Cy3-labelled cRNA from each of the five patients was mixed with the same amount of reverse color Cy5-labelled cRNA from the corresponding control. All experiments were also dye swapped with reverse colouring. The mixed labeled cRNA was hybridized onto Agilent`s 60-mer Human 1A (V2) oligo microarrays (G4110B). The hybridization of the cRNA labeled targets to the glass slide was done according to Agilent`s 60-mer oligo microarray processing protocol, version 4.1 (Agilent Technologies), and the slides were scanned using the Agilent Micro Array Scanner, version G2505B (Agilent Technologies). The raw data were loaded into the public domain MIAME-compliant database BASE, version 1.2.10 [4,5].

Feature extraction was done with the Agilent G2567AA Feature Extraction Software, version 7.5 (Agilent Technologies) with Linear & LOWESS normalization using default parameter settings. All control spots and all spots with intensities below 300 in either channel were flagged.

For finding the differentially expressed genes, statistical analyses of the normalized data were done using Bayesian ANOVA for Microarrays, BAMarray ([www.bamarray.com](http://www.bamarray.com/)). In order to use the BAMarray software an expression matrix was made. To get rid of the dye effect the mean of the two values obtained from each of the two experiments in the corresponding dye swap were computed for each case-control pair and gene. If one of the two values was missing the mean value was set equal to the non-missing value in the pair. If both values were missing, the mean value was also missing. After removing genes with data from only one or zero case-control pairs, missing values were imputed using the K-nearest neighbor imputation method in the Significance Analysis of Microarrays (SAM) statistical software (www-stat.stanford.edu/~tibs/SAM). The differentially expressed genes were then found from the resulting expression matrix using the BAMarray software.

To reduce the number of genes chosen for further analysis and presentation, strict selection criteria were applied. More precisely, ratio analysis of the normalized data combining the dye swapped experiments was performed using the Rosetta Luminator software, version 2.0 ([www.rosettabio.com](http://www.rosettabio.com/)). The software was set to include only genes with signal intensities > 300. Furthermore, a P value <0.01 was set for the probability that a gene was differentially expressed between case and control and classified as a signature gene. Finally, only the most differentially expressed signature genes (signature genes with mean fold changes >+2.50 or <-2.50) were included for further analysis. Of the differentially expressed genes found by the BAMarray method, only those also occurring in the list of genes found by the Rosetta Luminator software were included in the list of genes selected for final presentation.

The final gene list was annotated with GeneBank accession numbers and classified with gene ontology terms as given by tools from the Norwegian Microarray Consortium (NMC) gene database Genetools ([www.genetools.no](http://www.genetools.no/)).

**QRT-PCR study**

Based on the microarray results, we evaluated some of the most differentially expressed genes with QRT-PCR on TaqMan Low Density Array (TLDA) cards in a new study with RNA samples from the other 13 hospitalised infants. We also used this method to validate the gene expression levels found for the five male infants in the microarray experiment. RNA samples from four of the five one year old children were chosen as a pooled exogenous control in all studies using TLDA cards.

In order to identify the most stable endogenous control an experiment with several housekeeping genes was undertaken. The genes were spotted on a TaqMan Human Endogenous Control Plate (Applied Biosystems) and tested on RNA samples from four of the five hospitalised infants and corresponding controls. The plate evaluates the expression of eleven housekeeping genes in total RNA samples using a two-step, reverse transcription–polymerase chain reaction. The plate also features a unique internal positive control (IPC) designed to detect the presence of PCR inhibitors in test samples. Beta-glukuronidase (GUSB) was the most stabile candidate gene in all samples and therefore selected as endogenous control in the further studies (data not shown).

Custom-made TLDA cards (Applied Biosystems) was used to perform QRT-PCR. Each card included some of the genes most differentially expressed in the microarray experiment and our selected endogenous control. The genes were preconfigured in a 384-well format and spotted on a microfluidic card with 3 replicates per gene. RNA amplification of the samples was performed with MessageAmp^TM^ aRNA Amplification Kit (Ambion) and the protocol was followed including synthesis of cDNA. Loading the Micro Fluidic Cards, creating Micro Fluidic Card documents and Performing Micro Fluidic Card Runs were done according to the User Guide (ABI PRISM 7900HT, Sequence Detector system and SDS Enterprise Database).

The data recorded when running the TLDA cards were quantified using the SDS 2.1 software package (Applied Biosystems) and results from each card was quantified using the ∆∆ Ct method, where one sample was designated as exogenous control and to which all other samples were analyzed [6,7]. Briefly, ΔCt represents the threshold cycle (Ct) for each gene minus that of the endogenous control, and ΔΔCt represents the ΔCt for each gene minus that of the exogenous control. Relative quantification (RQ) of the genes in a sample was determined using the equation 2^-∆∆ Ct^, and for which the relative quantity equals one for the genes in the exogenous control sample. Therefore, every other sample is expressed relative to this control sample. Analysis of the RQ results was done by automatic determination of baseline and threshold, automatic outlier removal and RQ Max/Min Confidence level set at 95%.

References

1. Fleige S, Pfaffl MW: **RNA integrity and the effect on the real-time qRT-PCR performance.** *Mol Aspects Med* 2006, **27:** 126-139.

2. Ebekian B, Carballal G, Cerqueiro C, Avila M, Salomon H, Weissenbacher M: **[Serologic response measured by complement fixation in children with acute respiratory infection].** *Rev Argent Microbiol* 1990, **22:** 62-67.

3. Rainen L, Oelmueller U, Jurgensen S, Wyrich R, Ballas C, Schram J *et al*.: **Stabilization of mRNA expression in whole blood samples.** *Clin Chem* 2002, **48:** 1883-1890.

4. Brazma A, Hingamp P, Quackenbush J, Sherlock G, Spellman P, Stoeckert C *et al*.: **Minimum information about a microarray experiment (MIAME)-toward standards for microarray data.** *Nat Genet* 2001, **29:** 365-371.

5. Saal LH, Troein C, Vallon-Christersson J, Gruvberger S, Borg A, Peterson C: **BioArray Software Environment (BASE): a platform for comprehensive management and analysis of microarray data.** *Genome Biol* 2002, **3:** SOFTWARE0003.

6. Abruzzo LV, Lee KY, Fuller A, Silverman A, Keating MJ, Medeiros LJ *et al*.: **Validation of oligonucleotide microarray data using microfluidic low-density arrays: a new statistical method to normalize real-time RT-PCR data.** *Biotechniques* 2005, **38:** 785-792.

7. Livak KJ, Schmittgen TD: **Analysis of relative gene expression data using real-time quantitative PCR and the 2(-Delta Delta C(T)) Method.** *Methods* 2001, **25:** 402-408.
